# Relationship between medical students’ perceived instructor role and their approaches to using online learning technologies in a cloud-based virtual classroom

**DOI:** 10.1186/s12909-022-03604-3

**Published:** 2022-07-19

**Authors:** Rong Wang, Jiying Han, Chuanyong Liu, Lixiang Wang

**Affiliations:** 1 Department of Physiology and Pathophysiology, School of Basic Medical Sciences, Shandong University, Jinan, China; 2 School of Foreign Languages and Literature, Shandong University, Jinan, China; 3 Department of Pharmacology, School of Basic Medical Sciences, Shandong University, Jinan, China

**Keywords:** Medical faculty, Online learning technologies, Medical students, COVID-19, Distance learning

## Abstract

**Background:**

Students can take different approaches to using online learning technologies: deep and surface. It is important to understand the relationship between instructor role and student approaches to using online learning technologies in online learning settings supported by cloud computing techniques.

**Methods:**

A descriptive, cross-sectional study was conducted to analyze the relationships between medical students’ perceptions of instructor role (instructor support, instructor-student interaction, and instructor innovation) and students’ approaches to using online learning technologies in cloud-based virtual classrooms. A 25-item online questionnaire along with a sheet with basic demographic was administered to all medical students at Qilu Medical Schools of Shandong University China. Overall, 213 of 4000 medical students (5.34%) at the medical school participated in the survey.

**Results:**

The results showed high levels of medical students’ perceived instructor support, instructor-student interaction and instructor innovation. Most students adopted the deep approaches to using online learning technologies. Instructor support, instructor-student interaction and innovation were positively related to students’ deep approaches to using online learning technologies. Instructor support was negatively related to students’ surface approaches to using online learning technologies.

**Conclusions:**

The relationship between instructor role (instructor support, instructor-student interaction and instructor innovation) and students’ approaches to using online learning technologies highlight the importance of instructor support and innovation in facilitating students’ adoption of desirable approaches to learning from the application of technologies.

## Introduction

Students’ approaches to learning (SAL) are defined as ‘a composite of a motive and an appropriate strategy’ according to Biggs [1], and he categorized two main types of approaches to learning— surface and deep [2]. The key difference is that deep approaches involve the intention to understand and create meaning from what is being learned, whereas surface approaches involve an intention to reproduce. Deep approaches to learning can lead to motivation and develop the ability of self-directed learning which is a key aspect of adult learning in present medical education. Medical students can combine reflective practice with a checklist to reduce medical errors and solve complex medical problems. With the increasing use of the internet in higher education, online learning technology is widely used to implement learning, such as presentations, resource downloads and quizzes before cloud computing [3]. For medical students, online learning supported by cloud computing techniques including learning management platforms, multimedia (electronic database), tools for collaborative authoring, scheduling, and communication could be perceived as useful for learning [4]. In online learning settings, student approaches to using online learning technologies, as additions to approaches to learning, have received increasing attention in higher education. According to the Student Approaches to Using Online Learning Technologies (SAOLT) Scale [3], deep approaches to using online learning technologies are meaningful, engaged and developed understanding. To review and cross-reference ideas between different sources, the approaches are ‘likely to stimulate critical thinking and new research pathways, while students adopting this approach are trying to connect the ideas in the course to the real-world experiences’ [3], which is effective usage of online learning technologies have become thoroughly penetrates any category of critical thinking especially such as gathering relevant information, evaluating the credibility of a source, and communicating effectively with others and these skills are assumed to spark research inspiration. Whereas surface approaches to using online learning technologies are predominantly reproductive and are not efficient usage of the technologies to engage in inquiry, which are ‘ tend to restrict the use of online learning technologies to minimize work, to only fulfill minimum course requirements and to try to avoid developing a meaningful online presence’ [3]. In blended learning settings, researches into student approaches to learning and to using online learning technologies has commonly identified the positive association between deep approaches and academic achievement, and the negative relationship between surface approaches and academic achievement [3, 5–7]. Meanwhile, effective approaches to online learning technologies could contribute to the students’ learning outcomes through their effect on students’ learning process [3, 8]. Therefore, adopting both deep approaches to learning and to using online learning technologies, is imperative to be a reflective, self-directed medical practitioner. However, current knowledge about how to effectively help the medical students to develop deep approaches to online learning technologies remains very limited.

The concept of student approaches to learning is context and task-dependent [9–11]. The role of instructors, one of the key elements of learning contextual factors, has been acknowledged as a significant factor influencing the learning approaches adopted by students [11]. Studies exploring instructor role in student learning suggest that instructor role is mainly assessed in terms of instructional support [12, 13], instructor scaffolding strategies such as instructor-student interaction [14, 15], and instructor innovation [16]. These studies indicate that instructor role in online learning environments facilitates students’ understanding of the knowledge and encourages student engagement by utilizing various facilitation strategies and teaching tools supported by the cloud technique [14, 15, 17]. In online learning environments, instructional support refers to the students’ perceived instructional guidance from instructors, which includes providing relevant resources, timely feedback and explanation, and correcting students’ misunderstandings [12, 15, 16]. Instructor-student interaction is one type of three types interactions: teacher-student interaction (e. g. immediate and targeted feedback), peer interaction and student-content interaction. Instructors’ implementation of strategies to promote learner-instructor interaction has been identified as a driving force for promoting students’ motivation [12, 14]. Instructor innovation involves the new course design, unusual class activities and teaching techniques employed by instructors in order to meet the challenges faced by today’s education [18]. Although a considerable number of studies have been conducted to explore the relationship between instructor role and student approaches to learning (SAL) in traditional and blending learning settings across a range of disciplines, such as science [19], nursing [20], and medicine [21], an integrated understanding of instructor role and student approaches to using online learning technologies (SAOLT) have received relatively less attention amongst medical students in a cloud-based virtual classroom. The understanding of the relationship between instructor role and student approaches to using online learning technologies, would help instructors design and create an effective online learning environment and make effective use of the educational tools supported by cloud computing techniques.

As a consequence of the sudden outbreak of COVID19 in mainland China, the traditional face-to-face or blending teaching activities in higher education have been replaced by cloud-based virtual classrooms since 2020, leading to a shift from ‘forced remote learning’ to ‘the new normal’ online learning environments [22, 23]. The role of instructor is one of the important research branches of the learning environment, and it is a significant determinant of students’ learning outcomes. Since instructors have been found to use different teaching strategies for different learning environments [24], the contribution of this study is to provide evidence about instructor role and SAOLT in a specific context such as a cloud-based virtual classroom within a medical student sample. Within such a changed learning context, how do medical students perceive the instructor’s role, and what is the predominant learning approach to using online learning technologies amongst medical students? How do instructors influence medical students’ choices of learning approaches to using online learning technologies? An integral understanding of how various learning environmental factors may affect medical students’ learning approaches to using online learning technologies is crucial to create a favorable remote learning environment. The present study aims to explore the characteristics of and relationship between medical students’ perceived instructor role and approaches to using online learning technologies in a cloud-based virtual classroom in mainland China.

## Methods

### Ethical approval

The present study involving student participants was reviewed and approved by the University of Shandong Research Ethics Committee. All participants provided their signed consent to participate in this study.

### Participants

The research is based on data collected two months after the onset of synchronous online learning. To explore the relationship between medical students’ perceived instructor role and their approaches to using online learning technologies in the new learning environment, an online questionnaire was administered to all of the 4 thousand undergraduates of Qilu Medical School of Shandong University in China, though it was not mandatory for students to complete the questionnaire. About 213 students responded to this online survey, and the response rate is 5.34%. They are attending online courses where all the subjects are taught through a cloud-based virtual classroom such as Rain Classroom or Tecent Classroom. The cohort of 213 medical students who entered a first-year medical course in 2016–2019. Amongst 213 students (mean age = 21.14 ± 2.32 years), 152 (71.4%) were female and 61 (28.6%) were male. 20.7% of participants were freshmen, 22.5% were sophomores, 5.6% were juniors and 51.2% were seniors.

### Measures

The online questionnaire used in this study had two sections and was comprised of 25 items (see Table 1). The first section consisted of three measures used to assess participants’ perceptions of instructor role in three dimensions: instructional support (six items of Lee et al.’s study [25], instructor-student interaction (five items of Ali et al.’s study [26]) and instructor innovation (four items adapted from College and University Classroom Environment Inventory (CUCEI) [27] and the study of Johnson et al. [28]). The second section included 10 items from the questionnaire based on student approaches to online learning technologies questionnaire (SAOLT) [3], which measures student approaches to using online learning technologies. The SAOLT was built on the theoretical framework of the student approach to learning including qualitatively different approaches to learning: deep approaches which are well understood, or surface approaches which are mainly reproductive. Six and four items were used to represent deep and surface approaches to online learning technologies construct, respectively. All items were slightly modified to indicate the online learning environment, and all items were scored on a 5-point Likert-type scale ranging from 1 “strongly disagree” to 5 “strongly agree”. In this study, the reliabilities for the instructor role, deep and surface approach scales were generally above 0.80 (See Table 2 for details).

**Table 1:** Factor analysis of the questionnaire used in this study

The KMO and Bartlett’stest in the section	Variable (number of items)	Item	Factor				
			1	2	3	4	5
The role of instructors (KMO = 0.92, Bartlett test ofsphericity: χ2(105) = 1715.70, *p* < 0.00, total varianceexplained = 67.19%)	Instructor support (4)	The course goals/objectives were clearly outlined	**.70**				
		I knew what I was expected to accomplish eachweek	**.79**				
		The instructors provided clear instructions forassignments and quizzes	**.78**				
		The courses provided relevant resources	**.58**				
	Instructor-student interaction (7)	The feedback on the assignments was helpful		**.59**			
		I felt that I could ask any questions regarding thecourse materials to the instructors		**.67**			
		The instructors encouraged me to become activelyinvolved in the course discussions		**.61**			
		The instructors provided me feedback on my workthrough comments		**.72**			
		I was able to interact with the instructors during thecourse discussions		**.73**			
		The instructors treated me individually		**.70**			
		The instructors informed me about my progressperiodically		**.68**			
	Instructor innovation (4)	The instructors adopted different teaching methodsfrom those in face-to-face courses			**.77**		
		The instructors designed new online learningactivities to get us involved			**.84**		
		The instructors used various and innovativeteaching methods in the online courses			**.87**		
		The instructors assigned different learning tasks inonline courses			**.82**		
Student Approaches to Using Online Learning Technologies (KMO = 0.89, Bartlett test of sphericity: χ2(45) = 1626.58, *p* < 0.001, total variance explained = 76.35%)	Deep approach to online learning technology support(6)	I find I use the online learning technologies in thiscourse to further my research into a topic				**.88**	
		I spend time using the online learning technologiesin this course to develop my knowledge on keytopics				**.91**	
		I try to use the online learning technologies in thiscourse to achieve a more complete understanding ofkey concepts				**.90**	
		I find interacting with online learning technologies inthis course promotes deeper understanding of keyideas				**.93**	
		I try to use the online learning technologies in thiscourse to communicate with other participants totest my ideas				**.84**	
		I find using the online learning technologies in thiscourse help me to develop my critical thinking				**.86**	
	Surface approach to online learning technology (4)	I restrict my use of online learning technologies inthis course to do as little as possible					**.79**
		I do not use the online learning technologies in thiscourse to enable me to achieve my goals					**.86**
		I only use the online learning technologies in thiscourse to fulfil course requirements					**.82**
		I do not find using online technologies in this coursehelps me to understand things more deeply					**.65**

**Table 2:** Descriptive statistics and reliabilities of the variables (*N* = 192)

	**Instructor support**	**Instructor-student interaction**	**Instructor innovation**	**Deep approach**	**Surface approach**
***M***	3.81	3.94*	3.75	3.51	2.60*
***SD***	0.72	0.57	0.76	0.97	0.85
***Cronbach’s α***	0.80	0.88	0.90	0.96	0.82

Note. Measured on a scale from 1 (disagree) to 5 (agree), * *P* < 0.05.

### Data analysis

The statistical analysis of quantitative responses was carried out by SPSS 23.0. Cronbach’s α reliability coefficients were computed to determine the reliability of the sub-scales used in this study: instructional support, instructor-student interaction, instructor innovation, deep and surface approaches to using online learning technologies. Exploratory factor analysis (EFA) was performed to determine the factor structure. Paired t-tests or repeated measure one-way ANOVA were used to compare if there was a significant difference within the mean scores of the students’ perceptions of instructor role and learning approaches. With reference to a previous study on evaluating students' learning approaches, the higher mean score of each learning approach was used to determine the main learning methods used by students [29]. So the higher mean score was taken to be indicative of the individual’s predominant learning approach in this study. Multivariate analysis of variance (MANOVA) was employed to identify if the students’ perceptions of instructor role, deep and surface approaches to learning variables vary across gender, and grade. Correlations between instructor role and learning approach were analyzed by Pearson product-moment correlations and multiple linear regression analysis with collinearity diagnostics.

## Results

### Reliability and Exploratory Factor Analysis (EFA) for validity

As is shown in Table 2, Cronbach’s alpha coefficients for all factors ranged from 0.80 to 0.96 indicating that all of the constructs in this study had a “good” internal consistency.

Fifteen items (section-students’ perceptions of instructor role) and 10 items (section-SAOLT) were subjected to EFA with Varimax rotation using the principal component analysis. To verify whether the sampling was adequate and ideal for Factor Analysis, the Kaiser–Meyer–Olkin (KMO) measure and Bartlett test of sphericity were used. According to Table 1, the analysis results show that both KMO values for each section were higher than 0.85. Bartlett’s test of sphericity for two sections (*p* < 0.001) indicated that the factor analysis was appropriate. A factor analysis was performed to obtain item loading for each factor of the data. Factor loading of items less than 0.40 was used as a criterion to remove items. Table 1 shows the rotated factor analysis in which five factors and 25 items were obtained by EFA: aligning with an instructional support (Factor 1), an instructor-student interaction (Factor 2), an instructor innovation (Factor 3), a deep variable (Factor 4) and surface variable (Factor 5). Both 15 items (section-students’ perceptions of instructor role) and 10 items (section-SAOLT) with sufficient loadings explained more than 65% of the variance, respectively.

### Descriptive statistics

We removed from the analyses students who had adopted both deep and surface learning approaches (*n* = 21), 192 out of 213 students were left to be analyzed. Among 192 medical students for analyses, 140 (72.9%) were female and 52 (27.1%) were male. 20.8% of participants were freshmen, 20.8% were sophomores, 6.3% were juniors and 52.1% were seniors. Table 2 shows the descriptive statistics of the variables data for all medical students in the cloud-based virtual classrooms. The mean scores of three dimensions of instructor role were higher above 3.70, and that of instructor-student interaction scored the highest. Among the 192 students, 146 (76.0%) adopted the deep, and 46 (24%) adopted the surface as their predominant learning approach to using online learning technologies.

### Inferential analysis

The potential difference between mean scores of the subscales of instructor role was examined by a repeated measure one-way ANOVA. Huynh–Feldt correction test was used to compare the differences in mean scores for each sub-scale because the assumption of sphericity variance was violated. There was a significant difference between the mean scores of the three dimensions of instructor role (*F*_(1.66, 317.17)_ = 8.84, *p* < 0.001) Post-hoc Bonferroni test (See Table 3) indicated that of three dimensions of instructors’ role, the mean scores of instructor-student interaction were significantly higher than those of instructors’ support and instructor innovation. Results of Paired t-tests (See Table 4) showed there was a significant difference between the mean scores of deep approaches and surface approaches (*t*_(191)_ = 8.28, *p* < 0.001).

**Table 3:** Bonferroni post hoc test: The difference among the three dimensions of instructor role

**(I) Instructor role**	**(J) instructor role**	Mean Difference (I-J)	95% Confidence for interval Difference		Sig.
			Lower Bound	Upper Bound	
**Instructor support**	**Instructor innovation**	0.07	-0.07	0.20	0.72
**Instructor-student interaction**	**Instructor innovation**	0.19*	0.09	0.30	< 0.001
**Instructor support**	**Instructor-student interaction**	-0.13*	-0.22	-0.03	0.004

*P < 0.05

**Table 4:** Paired samples test: The difference between two types of learning approach

(I) Learning approach	(J) Learning approach	Mean Difference (I-J)	95% Confidence for Interval Difference		Sig.
			**Lower Bound**	**Upper Bound**	
**Deep approach to use online learning technologies**	**Surface approaches to use online learning technologies**	0.92**	0.70	1.14	(< 0.001)

**P < 0.001

MANOVA was employed to determine whether there were statistical differences in students’ perceptions of three dimensions of instructor role, deep and surface approaches to using online learning technologies among those with different demographic characteristics –- gender and grade. There was no significant main effect of gender and grade on instructor support.

### Relationships between students’ perceived instructor role and their learning approaches to using online learning technologies

Table 5 presents the results of the correlation analysis. Six significant relationships (*p* < 0.001) were noted with r values between − 0.24 and 0.67 indicating negative and positive relationships, respectively. No collinearity was detected between instructor support and instructor-student interaction or instructor innovation. The instructor support, instructor-student interaction and instructor innovation had significant positive or negative correlations to deep or surface learning approaches to using online learning technology, respectively. Table 6 is the results of the regression analysis. With regard to the instructor role on the approaches to using online learning technologies, the instructor support, instructor-student interaction and instructor innovation had a significant positive effect on the deep approach to using online learning technologies, and instructor support also had a significant negative effect on the surface approach to using online learning technologies.

**Table 5:** The correlation coefficients between instructor role and the learning approaches to use online learning technologies scales

		**Instructor support**	**Instructor-student interaction**	**Instructor innovation**	**Deep approach**	**Surface approach**
**Instructor support**	*r* (*p*-value)	1	0.67** (< 0.001)	0.45** (< 0.001)	0.59** (< 0.001)	– 0.45** (< 0.001)
**Instructor-student interaction**	*r* (*p*-value)		1	0.63** (< 0.001)	0.61** (< 0.001)	– 0.36** (< 0.001)
**Instructor innovation**	*r* (*p*-value)			1	0.62** (< 0.001)	– 0.24** (< 0.001)
**Deep approach**	*r* (*p*-value)				1	– 0.44** (< 0.001)
**Surface approach**						1

** *P* < 0.001

**Table 6:** Regression results of instructor role on approaches to use online learning technologies

Dependent variable	*R*^2^	Instructor support	Instructor-student interaction	Instructor innovation
**Deep approach**	0.52	0.31**	0.16*	0.38**
**Surface approach**	0.20	– 0.37**	– 0.10	-0.01

* *P* < 0.05, ** *P* < 0.001

## Discussion

Our first research question asked: In a cloud-based virtual classroom, how do medical students perceive the instructor role, and what is the predominant learning approach to using online learning technologies amongst medical students? Since the mean scores of all three dimensions of the instructor role were higher than 3.70, medical students were considered to endorse a certain amount of support and interactivity provided by their instructors, and instructors had a certain amount of innovations in a cloud-based virtual classroom. Meanwhile, most medical students agreed that they had adopted more deep approaches rather than surface approaches to learning in a cloud-based virtual classroom. Previous research indicated that providing effective instructional support and interactivity was particularly critical for the instructors in online learning environments [14, 16]. Therefore, in a cloud-based virtual classroom, medical students could perceive a satisfactory learning process supported by instructors who have made full use of various cloud-based technology, such as Tecent Docs, bullet subtitles for sending queries and recording video of a class in Rain Classroom, or We Chat by enhancing their behavioral and cognitive engagement in e-learning environment [17]. However, the results also indicated that students perceived a higher level of instructor-student interaction than that of instructor support and instructor innovation during the learning experience in a cloud-based virtual classroom. So, it is still a challenge for instructors and administrators how to improve effective and innovative teaching support by using cloud computing technology in cloud-based learning settings.

Results of the SOALT questionnaire showed that medical students adopted more deep rather than surface approaches to using online learning technologies in a cloud-based virtual classroom. This result is consistent with the results of previous research with medical students in blended learning environments [30]. The possible explanation could be due to the medical curricula reform made in China in response to the Ministry of Education’s requirements to cultivate more reflective and self-directed medical practitioners, which was accordant with the global trend towards encouraging deeper learning in medical education [31]. The main aim of medical curricula reform is to create a student-centered learning environment in which various teaching methods, unlike didactic pedagogies, are adopted to foster deep learning and understanding [32–34]. According to constructivist learning theory [34, 35], instructors may promote students’ deep approaches to learning by “ways of thinking about teaching and learning that emphasize student responsibility and activity in learning rather than content or what the teachers are doing” (Cannon and Newble, pp. 16–17) [35]. Given that students may develop their approaches to using online learning technologies as shown by their level of responses and activity, it could be possible for instructors to make students more engaged in learning and better encourage them to adopt deeper approaches to using online learning technologies, such as inquiry-based activities designed by the instructor via cloud-based education apps [36].

This study showed no significant difference in students’ perceptions of instructor role and approaches among those with different demographic characteristics such as gender and grade. The previous studies about the demographic characteristics are mixed and elusive [37–39]. Since students’ approaches to using online learning technologies are dependent on the learning environment and experience [3, 40], it is necessary to further investigate the impact of student demographics on the online learning process in a cloud-based classroom.

The second research question asked: How do instructors influence medical students’ choices of learning approaches to using online learning technologies in a cloud-based virtual classroom? This study revealed a significant positive relationship between instructor role and students’ deep approaches, and a significant negative contribution of instructor support to the students’ adoption of surface approaches in a cloud-based virtual classroom. The instructor innovation contributed more than instructor support or instructor-student interaction as a significant coefficient in the regression with deep approaches to using online learning technologies as the dependent variable.

The positive effect of instructor role on students’ adoption of deep approaches to use online learning technologies among medical students in a cloud-based virtual classroom, to our knowledge, is the first time to be reported. The results are similar to the findings of previous research into deep approaches to learning in blended learning environments [41]. To attain deeper learning in a cloud-based virtual classroom, it was more critical for instructors to offer proper guides on how to adopt deep approaches to using online learning technologies during learning process [3, 7, 14, 30]. The instructional guidance not only helps to deepen content understanding through the well-designed inquiry-based tasks etc. and promotes collective, cumulative and purposeful class interactions, but may also include the integration of content knowledge and online learning technologies in the online learning context, for example, how to develop and evaluate high-quality performance assessments via cloud-based education apps to have a deeper understanding of what kinds of tasks motivate thoughtful work, and how students think as they fulfilled the tasks. In this study, although students’ perception of instructor-student interaction was the highest level among the three, the contribution of it to enhancing students’ deep approaches to online learning technology was the smallest which may be due to the ineffective or low-quality class interactions. It is perhaps not surprising that the most positive correlations between instructor innovation and deep approaches to using online learning technologies, as instructors who keep pace with these new educational technologies could consider innovative and effective course design based on student needs, use various kinds of assessments and perform activities with personal characteristics supported by a good integration of content and cloud computing learning technologies [14, 42]. To stimulate students’ deep learning in online learning environments, the challenges of instructors’ innovation may include how to develop online adaptive expertise through inquiry-based tasks to find gaps and understand deviations in students’ knowledge and acquire new structures, explanations, and forms of interaction, and how to encourage them to express their own cognitive processes and analyze and self-evaluate their problem-solving. Furthermore, it is well known that assessment can be instructive [43]. Therefore, the other challenges of instructors’ innovation might include how to create an effective online assessments system that encourages the kinds of learning that are required to achieve the goals of career readiness.

The negative effect of instructional support on medical students’ adoption of surface approaches to using online learning technologies further supported the importance of instructional support to reduce student adoption of surface approaches. Compared with deep approaches, surface approaches to learning and to using online learning technologies are positively related to perceptions of unreasonable online course design and learning workload, and poor academic performance in blended learning settings [3, 44]. Therefore, instructor support could be the key to creating a high-quality online learning environment by providing proper instructional guidance mentioned above to boost deep approaches and reduce surface approaches to using online learning technologies.

Despite the low response rate of this study, the results are still encouraging and might be considered as representative because the demographic structure in terms of age and gender is consistent with current data on the broader medical undergraduate population in Chinese public universities. There are two possible reasons for the low response rate. The first one could be the general perception of lower student response rates of the online surveys compared to other survey methods such as paper-based surveys [45, 46]. The second one could be that medical students were less interested in the research topic of this survey because they thought the pure synchronous online teaching model was only a temporary emergency tool. Therefore, many students lacked the enthusiasm to respond to the questionnaires distributed by QR codes. This can be explained by the results of previous studies on the high correlation between survey response rates and participants' research interests [47].

This finding contributes to the body of literature by examining the relationship between the instructor role and students' approaches to using online learning technologies in cloud-based learning environments. Furthermore, it contributes to our understanding of why instructor support and innovation could be related to improving the quality of cloud-based online teaching and learning.

### Limitations

The low response rate is one of the limitations of this study. The small sample group could increase the risk of receiving skewed results that don’t fairly represent the rest of the students. To solve the issue of low response rate of the online surveys, higher response rates could be achievable by using multiple contacts, and teacher-student communication and incentives in future online surveys [45]. The present study might offer some insight into the characteristics of and relationships between instructor role and students’ approaches to using online learning technologies in a cloud-based virtual classroom in mainland China. Some limitations should be noted as indications for future work. First, the implications of our work for actual learning are limited. Although Ellis et. al. demonstrated the positive correlation between SAL and SOALT in a blended environment [3], further studies should be needed on the associations and interplay among SAL, SOALT and actual learning outcomes at the level of dependent variables in a specific context. In addition, the results of this study were based on the self-report data, therefore, some biases and limitations such as social desirability or introspective ability are inevitable. To improve our ability to interpret the effects of instructors’ role, SAL and/or SOALT on actual learning outcomes, a mixture of data sources—including self-report data and an evaluation of the final assignment—will be needed. Based on Biggs’s 3P Model of Student Learning theory [48], the triadic reciprocal causation among Presage, Process and Product suggests that there are several possible models of the interrelationships amongst the role of the instructor, students’ learning approaches including usage of online technology, and learning outcomes. Accordingly, further investigation of the interrelationships among the three variables is required to help teachers to design high-quality inquiry-based activities which make much more efficient use of the Internet. Second, three dimensions of the instructor role in this study were examined (instructor support, instructor-student interaction, and instructor innovation). It is also essential to determine how students’ approaches to using online learning technologies may change when instructors perform more different roles, such as boosting peer interaction. Last but not least, further study may consider a longitudinal research design to determine the consistent causation between these variables.

## Conclusion

The results showed medical students appreciated instructor support, instructor-student interaction, and instructor innovation during the learning process, most students adopted the deep as their predominant learning approach to using online learning technologies in a cloud-based virtual classroom. Medical students associated greater instructor support, instructor-student interaction, and innovation with greater self-reported use of deep learning online tools, and instructor support had significant negative effects on the adoption of students’ surface approaches to using online learning technologies. This study indicates that instructor-student interaction still needs to be improved, and instructor support and innovation could be more important in facilitating students’ adoption of desirable approaches to online learning technologies in a synchronous online learning setting.

## Data Availability

The dataset used during the study is available from the corresponding author on reasonable request.
